# First person – Tamsin Samuels

**DOI:** 10.1242/bio.051698

**Published:** 2020-05-04

## Abstract

First Person is a series of interviews with the first authors of a selection of papers published in Biology Open, helping early-career researchers promote themselves alongside their papers. Tamsin Samuels is first author on ‘ Neuronal upregulation of Prospero protein is driven by alternative mRNA polyadenylation and Syncrip-mediated mRNA stabilisation’, published in BiO. Tamsin conducted the research described in this article while a DPhil (PhD) student in Professor Ilan Davis's lab at the Department of Biochemistry, University of Oxford, Oxford, UK. She is now a postdoc in the lab of Dr Felipe Karam Teixeira at the Department of Genetics, University of Cambridge, Cambridge, UK, investigating the regulation of stem cells in their native tissue environment.


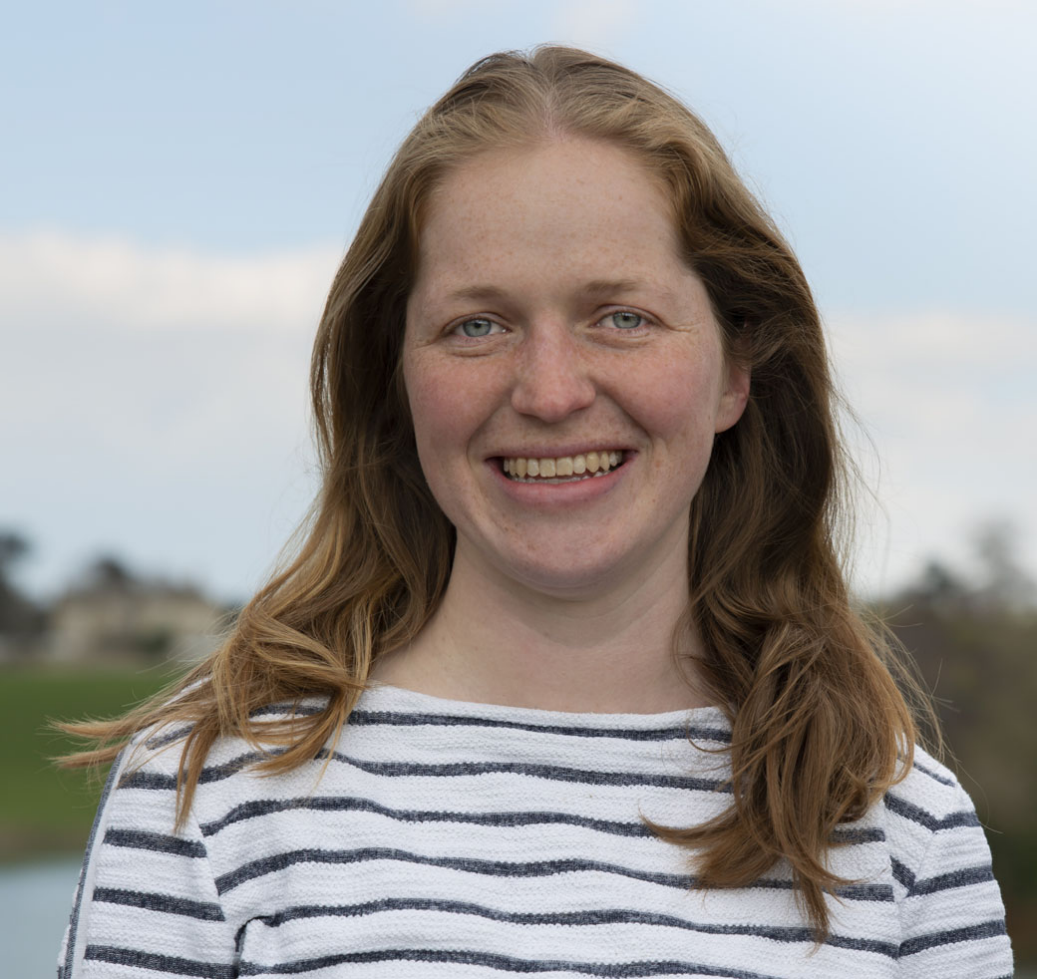


**Tamsin Samuels**

**What is your scientific background and the general focus of your lab?**

I completed my undergraduate degree in Natural Sciences, specialising in Biochemistry, and in my final year I completed a research project in Simon Bullock's lab at the Laboratory of Molecular Biology, working on RNA localisation in fly neurons. This project introduced me to the fruit fly as a model system, including the huge toolkit of genetic manipulations and the potential for subcellular microscopy. I worked on the project published here during my PhD in Ilan Davis' group at the University of Oxford. The lab focuses on post-transcriptional regulation (localisation, stability and translation of the mRNA intermediate) in the fruit fly nervous system. I have been interested in the regulation of the neural stem cells and their differentiating daughter cells.

**How would you explain the main findings of your paper to non-scientific family and friends?**

All the cells of the brain are produced by a small number of neural stem cells that divide repeatedly. The daughter cells undergo a process of specialisation into neurons that is driven by a regulatory factor called Prospero. We wanted to understand the regulation of Prospero, and how its levels are increased during the specialisation process. Genes are copied into an intermediate called messenger RNA (mRNA), which is then transported around the cell and then translated to produce a specific protein. The mRNA encodes the protein but also includes regulatory sequences that determine the localisation and stability of the mRNA. We found that *prospero* is expressed in several different mRNA version (or isoforms), including different regulatory sequences. One of the isoforms is much longer than the others, and we found that it is specifically stabilised by binding to a regulatory protein, Syncrip, in the specialising neurons. Increasing the stability of the mRNA means that more Prospero protein is produced from this isoform. The shorter isoforms aren't stabilised by Syncrip. Therefore the expression pattern of the different isoforms controls the amount of Prospero protein produced.

**What are the potential implications of these results for your field of research?**

Our work examines a mechanism through which a gene can be differently regulated in different cell types. Different mRNA isoforms of *prospero* have different mRNA stabilities and therefore the choice of isoform expression is a critical regulatory step. This finding highlights the complex multiple layers of gene regulation.

**What has surprised you the most while conducting your research?**

During the project we used CRISPR/Cas9 genome editing to remove one isoform of *prospero* (with a really long untranslated extension). However, making this deletion was surprisingly difficult due to the complex isoform structures and unexpected additional isoforms. Perhaps this flexibility makes the system more resilient to perturbations.

“… making this deletion was surprisingly difficult due to the complex isoform structures and unexpected additional isoforms. Perhaps this flexibility makes the system more resilient to perturbations.”

**What's next for you?**

I have moved to the University of Cambridge and joined Felipe Karam Teixeira's group in the Genetics department as a postdoc. I am still interested in RNA regulation in stem cells, but have moved from the neural stem cells to the germline stem cells in the fruit fly ovary.

**Figure:**
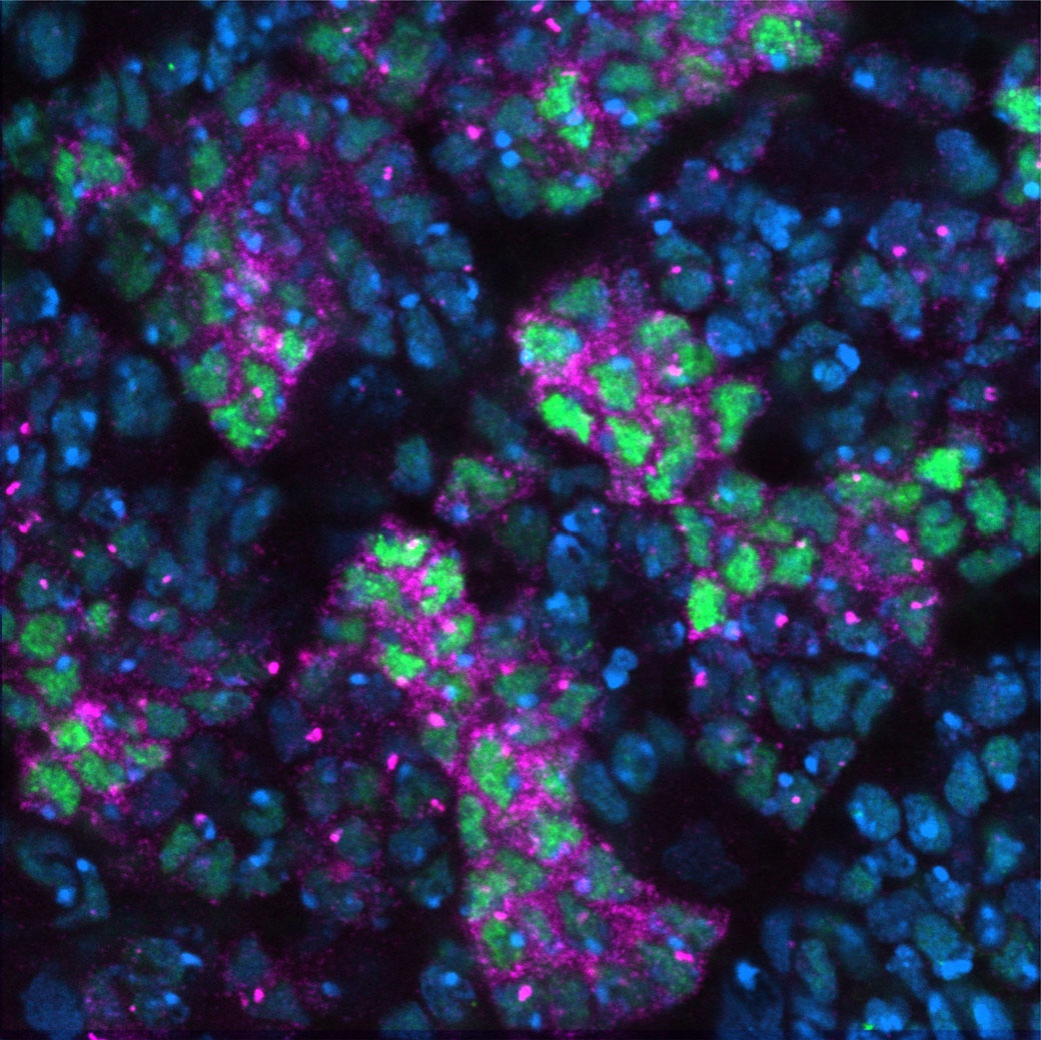
**A larval fruit fly brain stained for DNA (DAPI, blue) and prospero protein (green) and mRNA (magenta).**
